# Chelation-Assisted Iron-Catalyzed C–H Activations: Scope and Mechanism

**DOI:** 10.1021/acs.accounts.3c00294

**Published:** 2023-12-20

**Authors:** Jiayu Mo, Antonis M. Messinis, Jinlian Li, Svenja Warratz, Lutz Ackermann

**Affiliations:** † Institut für Organische und Biomolekulare Chemie, Georg-August-Universität Göttingen, Tammannstraße 2, 37077 Göttingen, Germany; ‡ School of Pharmacy, Guangxi Medical University, Shuangyong Road 22, 530021 Nanning, P. R. China; § WISCh (Wöhler-Research Institute for Sustainable Chemistry), Georg-August-Universität Göttingen, Tammannstraße 2, Göttingen 37077, Germany

## Abstract

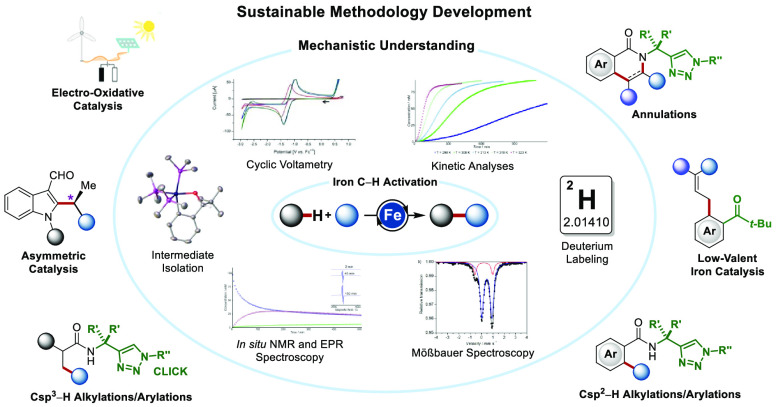

To improve the resource economy of molecular syntheses, researchers have developed strategies to directly activate otherwise inert C–H bonds, thus avoiding cumbersome and costly substrate prefunctionalizations. During the past two decades, remarkable progress in coordination chemistry has set the stage for developing increasingly viable metal catalysts for C–H activations. Despite remarkable advances, C–H activations are largely dominated by precious 4d and 5d transition metal catalysts based primarily on palladium, ruthenium, iridium, and rhodium, thus decreasing the inherent sustainable nature of the C–H activation approach. Therefore, advancing catalytic reactions based on Earth-abundant and less toxic 3d transition metals, especially nontoxic and inexpensive iron, represents a desirable and attractive alternative. While research had previously focused on 8-aminoquinoline directing groups in C–H activations, we have devised easily accessible, tunable, and clickable triazoles, which feature widespread applications in bioactive compounds and drugs, among others, as peptide isosteres. Thus, in contrast to other directing groups, the triazole group is a highly desirable structural motif and functions as a bioisostere in medicine and biology, where it is exploited to mimic amide bonds.

This Account summarizes the evolution of chelation-assisted iron-catalyzed C–H activations via C–H, C–H/N–H, and C–H/N–H/C–C bond cleavages, with a topical focus on the most recent contributions of our team. Thus, the triazole-enabled iron catalysis has surfaced as a transformative platform for a large variety of C–H transformations, including arylations, alkylations, alkenylations, allylations, annulations, and alkynylations, achieved through C–H activations with organometallic reagents, organohalides, alkynes, alkenes, allenes, and bicyclopropylidenes among others. Consequently, we developed widely applicable methods for the versatile preparation of decorated arenes and heteroarenes, providing access to benzamides, isoquinolones, pyrrolones, pyridones, phenones, indoles, and isoindolinones, among others. Most of these reactions employed 1,2-dichloroisobutane (DCIB) as an oxidant. Notably, chemical-oxidant-free strategies were also developed, with the major breakthroughs being the use of internal oxidants in oxidative annulations, the use of resource-economic electrocatalysis, and the development of well-defined iron(0)-mediated catalysis. In addition, a highly enantioselective inner-sphere C–H alkylation of (aza)indoles was developed by designing novel remotely decorated N-heterocyclic carbene ligands with dispersion energy donors. In addition, detailed mechanistic experiments including kinetic analyses, intermediate isolation, Mößbauer spectroscopy, and computation provided strong support for the mode of catalysis operation, enabling unprecedented C–H activations. Thereby, low-valent iron catalysts paved the way toward weakly coordinating ketones and enantioselective iron-catalyzed C–H activations through organometallic intermediates.

## Key References

- MoJ.; MessinisA. M.; OliveiraJ. C. A.; DemeshkoS.; MeyerF.; AckermannL.Iron-Catalyzed Triazole-Enabled C–H Activation with Bicyclopropylidenes. ACS Catal.2021, 11, 1053–1064.^1^*The first example of tunable iron-catalyzed C–H/C–C activation, leading to the discovery of rare C–F/C–H activations in iron catalysis*.
- ZhuC.; StangierM.; OliveiraJ. C. A.; MassignanL.; AckermannL.Iron-Electrocatalyzed C–H Arylations: Mechanistic Insights into Oxidation-Induced Reductive Elimination for Ferraelectrocatalysis. Chem.—Eur. J.2019, 25, 16382–1638931658385 10.1002/chem.201904018PMC6972497.^2^*The first iron-electrocatalyzed C–H activation was devised*. *Here, ferraelectro-catalyzed C–H arylations under mild conditions were accomplished with ample scope, exploiting electricity as a benign oxidant*.
- MessinisA. M.; FingerL. H.; HuL.; AckermannL.Allenes for Versatile Iron-Catalyzed C–H Activation by Weak *O*-Coordination: Mechanistic Insights by Kinetics, Intermediate Isolation, and Computation. J. Am. Chem. Soc.2020, 142, 13102–1311132536163 10.1021/jacs.0c04837.^3^*A low valent iron-catalyzed C–H activation with allenes was developed with detailed mechanistic studies, leading to a highly regio- and chemoselective transformation*.
- LoupJ.; ZellD.; OliveiraJ. C. A.; KeilH.; StalkeD.; AckermannL.Asymmetric Iron-Catalyzed C–H Alkylation Enabled by Remote Ligand *meta*-Substitution. Angew. Chem., Int. Ed.2017, 56, 14197–1420110.1002/anie.20170907528922549.^4^*The first highly enantioselective iron-catalyzed C–H activation was developed by novel NHC ligands with dispersion energy donors*.

## 1 Introduction

**Figure 1:**
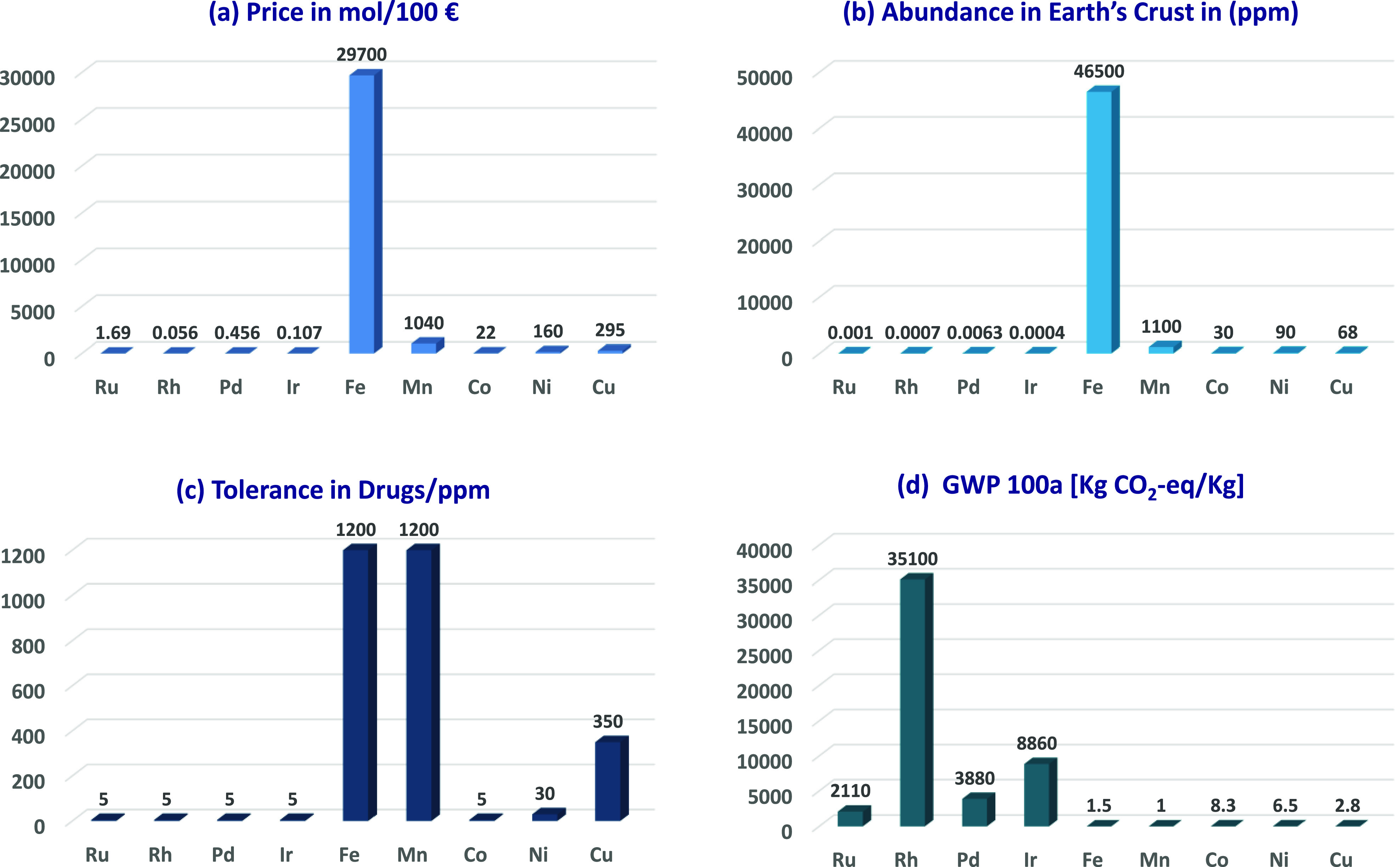
Advantages of iron. (a) Price comparison (mol/100 €) of iron with other commonly applied transition metals. (b) Natural abundance of common transition metals in the earth’s crust in ppm. (c) Tolerance of transition metals in drugs. *High tolerance; the exact amount could not be determined. (d) Global warming potential of metal production. Data were taken from refs (9, 10, 13, and 14).

The quest for sustainable approaches to access complex organic molecules constitutes a driving force for academic and industrial research. During the past decades, transition-metal catalysis has witnessed significant advances. Within this broad field, the transition-metal-catalyzed activation of otherwise inert C–H bonds is one of the most powerful and environmentally benign synthesis strategies.^5^ Hence, the unique potential offered by catalytic C–H activation to enhance the atom- and step-economy of organic syntheses has gained considerable momentum.^6^ Despite significant advances, sustainable, efficient, and selective^7^ C–H activations of structurally complex molecules with full stereoselectivity^8^ pose considerable challenges since they contain numerous C–H bonds with comparable dissociation energies. Specifically, significant progress was achieved with palladium, iridium, rhodium, and ruthenium catalysts in chelation-assisted C–H activations.^5,7,8^ These transition metals are expensive,^9^ scarce,^10^ and toxic (Figure 1a–c),^11^ thus decreasing the sustainable nature of the C–H activation approach. To address these sustainability aspects, 3d transition metals have been explored as environmentally benign alternatives to noble metals.^12^ Within the 3d transition metals, iron stands out due to its low cost,^9^ high natural abundance,^10^ viable trace metal impurities in pharmaceutical products,^13^ and low global warming potential (Figure 1d).^14^ Iron also features several oxidation states (ranging from −II to +VI) that can promote catalysis.^15^

In this context, the C–H activations examined herein proceed via three main mechanistic pathways, depending on the applied catalysis conditions and the nature of the directing group. Hence, when iron(II)/(III) precatalysts were used in combination with an external oxidant, such as dichloroisobutane (DCIB), and triazole as the directing group, a single electron oxidative iron(II)/iron(III)/iron(I) pathway was observed. C–H activation typically occurs via a σ-bond metathesis (DBM)/deprotonative metalation or a ligand-to-ligand hydrogen transfer (LLHT). When electrophilic substrates are used or in redox-neutral annulations, an iron(II) pathway is operative. In contrast, employing iron(0) species in catalysis in combination with weakly coordinating directing groups enables C–H activations via a low valent pathway that involves an oxidative addition to an iron(0) intermediate as the C–H activation step toward the formation of an iron hydride.

## 2 Triazoles as Efficient Directing Groups in Iron-Catalyzed C–H Activations

The major challenge in C–H activation lies in the wealth of C–H bonds with comparable bond dissociation energies. While the choice of catalyst and ligand governs the C–H activation efficiency, site-selectivity control can be achieved by chelation assistance, bringing the metal near the target C–H bond.^7^ Compared to the large variety of directing groups compatible with noble metal-catalyzed C–H activation,^7^ only a limited number have been identified to facilitate iron-catalyzed C–H activations.^16^ Therefore, discovering novel directing groups compatible with iron-catalyzed C–H activations is crucial for developing green and sustainable methodologies.

Early studies demonstrated the high potential of nitrogen heterocycles as good directing groups in iron-catalyzed C–H activation.^16^ Characteristic early examples are the iron-catalyzed C–H arylations of 2-phenylpyridine, where the importance of 1,2-dichloroisobutane (DCIB) as the oxidant was highlighted.^17^ Subsequent studies further exploited monodentate directing groups, advancing the field of iron-catalyzed C(sp^2^)–H arylations, alkylations, and alkenylations.^16^ Following studies by Daugulis on 8-aminoquinoline (8-AQ)-assisted C–H activation,^18^ this bidentate directing group for iron-catalysis was exploited by Nakamura,^19^ which expanded the range of transformations, including allylation, oxidative annulation, and amination.^16^ However, the development of iron-catalyzed C–H activations with 8-AQ as an auxiliary has been constrained by its reduced site-selectivity, restricted potential for structural modification, and need for its removal in the final product.^20^

**Scheme 1:**
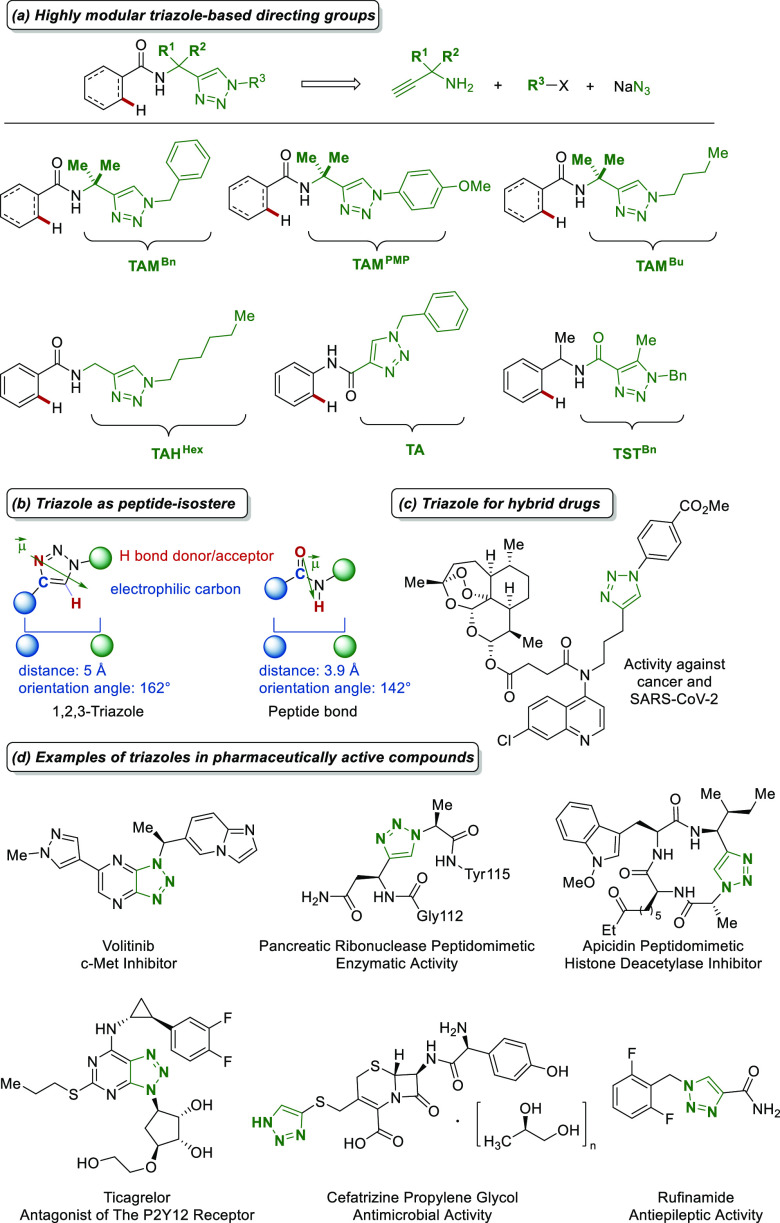
Highly Modular 1,2,3-Triazoles in Drug Discovery

Triazoles are an important family of nitrogen heterocycles due to their modularity (Scheme 1a, clickable assembly), accessibility,^21^ and bioactivity (Scheme 1b,c).^22^ Triazole’s structural similarity to other functional groups (Scheme 1b) enables seamless substitution in drug molecules, often leading to enhanced bioactivity and improved pharmacokinetic profiles toward safer and more effective therapeutic agents.^22a^ Replacing a peptide bond with a triazole can increase the peptide stability and conjugation with antibodies and is highly interesting for developing peptidomimetics.^22b^ According to previous reports by our group, triazoles were recognized as efficient directing groups in precious-metal-catalyzed C–H activations.^23^ Hence, we began to explore triazoles as directing groups in the iron-catalyzed C–H activation arena.

## 3 Triazole-Assisted Iron-Catalyzed C–H Activations

### 3.1 Arene C(sp^2^)–H Activations with Organometallic and Organohalide Substrates

**Scheme 2:**
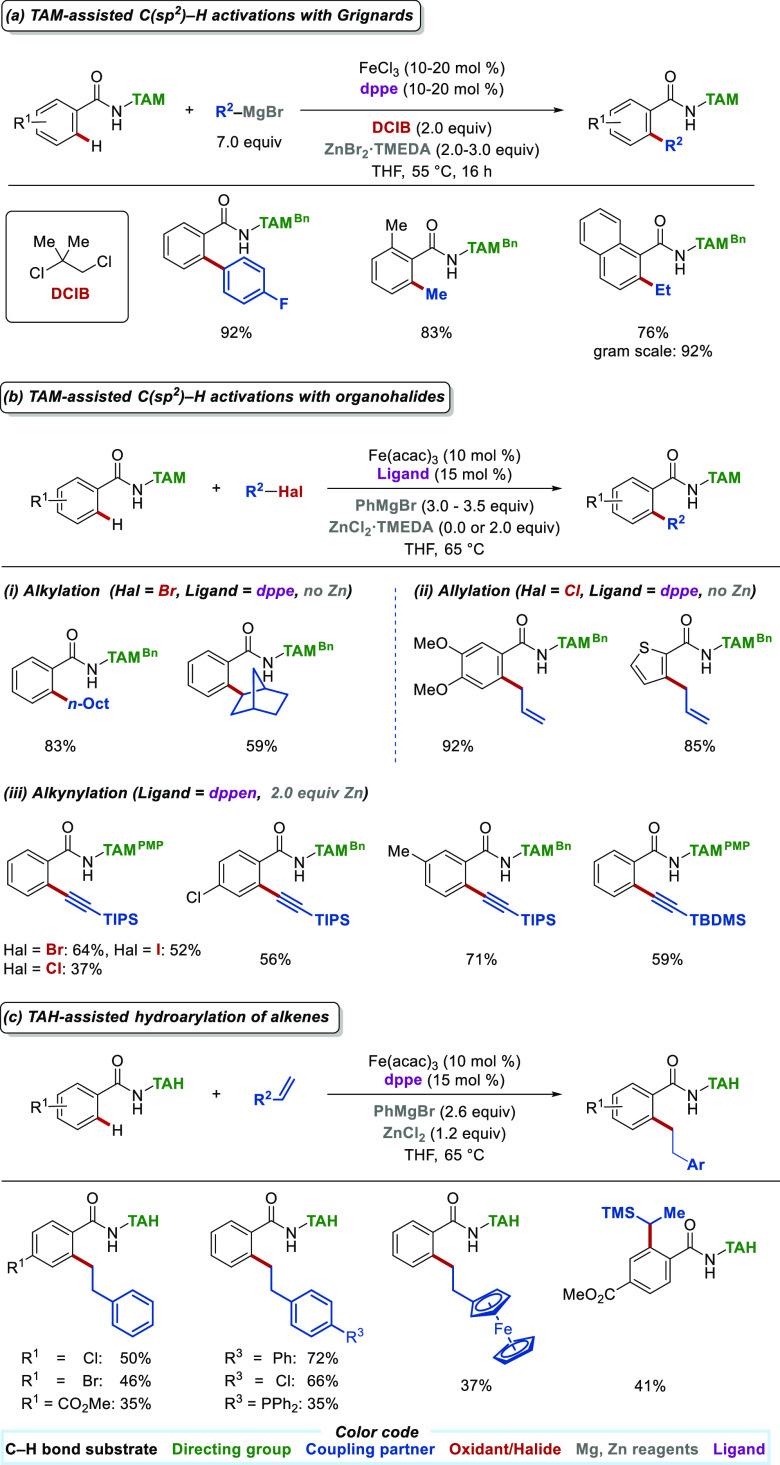
Summary of TAM- and TAH-Directed Iron-Catalyzed C(sp^2^)–H Activations with Organometallic and Organohalide Substrates

Given the properties of triazoles, we successfully exploited these structural scaffolds in iron-catalyzed C–H arylations^24^ and alkylations^25^ using Grignard reagents as coupling partners (Scheme 2a). Similar to methylations, arylations are of utmost importance since introducing aryl groups to a molecule increases molecular diversity and can enhance desirable molecular properties such as solubility, bioactivity, and toxicity.^26^ Here, triazole assistance enabled iron-catalyzed C(sp^2^)–H arylations with high efficiency and complete monoselectivity, overcoming chemoselectivity hurdles observed with other *ortho*-directing groups.^16^ Likewise, efficient alkylations^25^ were developed by suppressing undesired β-hydride elimination, opening the path toward triazole-assisted iron-catalyzed asymmetric C–H alkylations.^27^ Various iron salts can promote the transformation, with iron(III) salts being the most efficient. In contrast, the presence of oxidant and zinc salt is indispensable since the lack of either shuts down catalysis. Therefore, in line with previous reports on quinoline assistance,^28^ chlorinated hydrocarbons proved to be sufficient oxidants, with DCIB being optimal. As for the zinc salt, it is proposed to form *in situ* the putative active nucleophile Ar_2_Zn/MgBr_2_ from ArMgBr and ZnBr_2_·TMEDA. Additional ArMgBr is also required as a base to deprotonate the amide, while chelating phosphines such as 1,2-bis(diphenylphosphino)ethane (dppe) and 1,2-bis(diphenylphosphino)benzene (dppbz) were best at promoting the reaction.

While alkylations with Grignard reagents are possible (Scheme 2a),^25^ the requirement for the prior synthesis of the Grignard, its use in large quantities, and the need for external oxidants reduces reaction sustainability and ease of application. Therefore, alternative strategies with organic halides were developed.^16,29^ Here, the TAM triazole chelation assistance enabled iron-catalyzed C–H alkylations and allylations with organohalides as substrates toward alkylated and allylated TAM benzamides with good chemo- and regioselectivity (Scheme 2b).^30^ The reaction conditions mimic those used with Grignard reagents, with the biggest differences being the slow addition of the PhMgBr and the lack of the zinc salt. Not meeting the latter conditions results in low yields and undesired phenylated byproduct formation.^29^ This observation has mechanistic implications discussed at the end of this section. Notably, the 8-AQ directing group gave less than satisfactory results under otherwise identical reaction conditions.

Despite the importance of alkynes as versatile building blocks for organic synthesis, iron-catalyzed alkynylations with readily available haloalkynes have not yet been realized. Encouraged by our results on triazole-assisted C–H alkylation and allylation with halides,^30^ we devised a strategy for iron-catalyzed C–H alkynylations with bromoalkynes as the electrophile.^31^ Here, and in contrast to alkyl halides, homocleavage of the C–Br bond is more challenging, blocking the arylation pathway (Scheme 2a) and enabling alkynylation (Scheme 2b(iii)). Regarding the phosphine ligands, dppe, dppbz, and 1,2-bis(diphenylphosphino)ethene (dppen) were found to be suitable, with the latter being optimal. This demonstrates the high tolerance of this reaction type in phosphine ligands, with the main requirement being the use of bidentate aryl phosphines.

High atom economy is essential for sustainable development due to the minimal waste produced.^6^ Therefore, atom-economical iron-catalyzed C–H activations are highly sustainable and green methodologies for directly modifying organic substrates. Hence, TAH assistance enabled the development of highly atom economic hydroarylations of alkenes (Scheme 2c).^32^ The superiority of TAH over TAM is attributed to the Thorpe–Ingold effect. Importantly, the selectivity of the reaction was switchable depending on the nature of the olefin, with vinylsilanes resulting in Markovnikov products, providing a complementary selectivity compared to 8-AQ.^33^ The reaction conditions are similar to those used in alkynylations (Scheme 2b(iii)), with the crucial difference that the presence of TMEDA in the zinc salt switches catalysis off.

### 3.2 Mechanistic Investigations on the Arene C(sp^2^)–H Activation Systems

**Figure 2:**
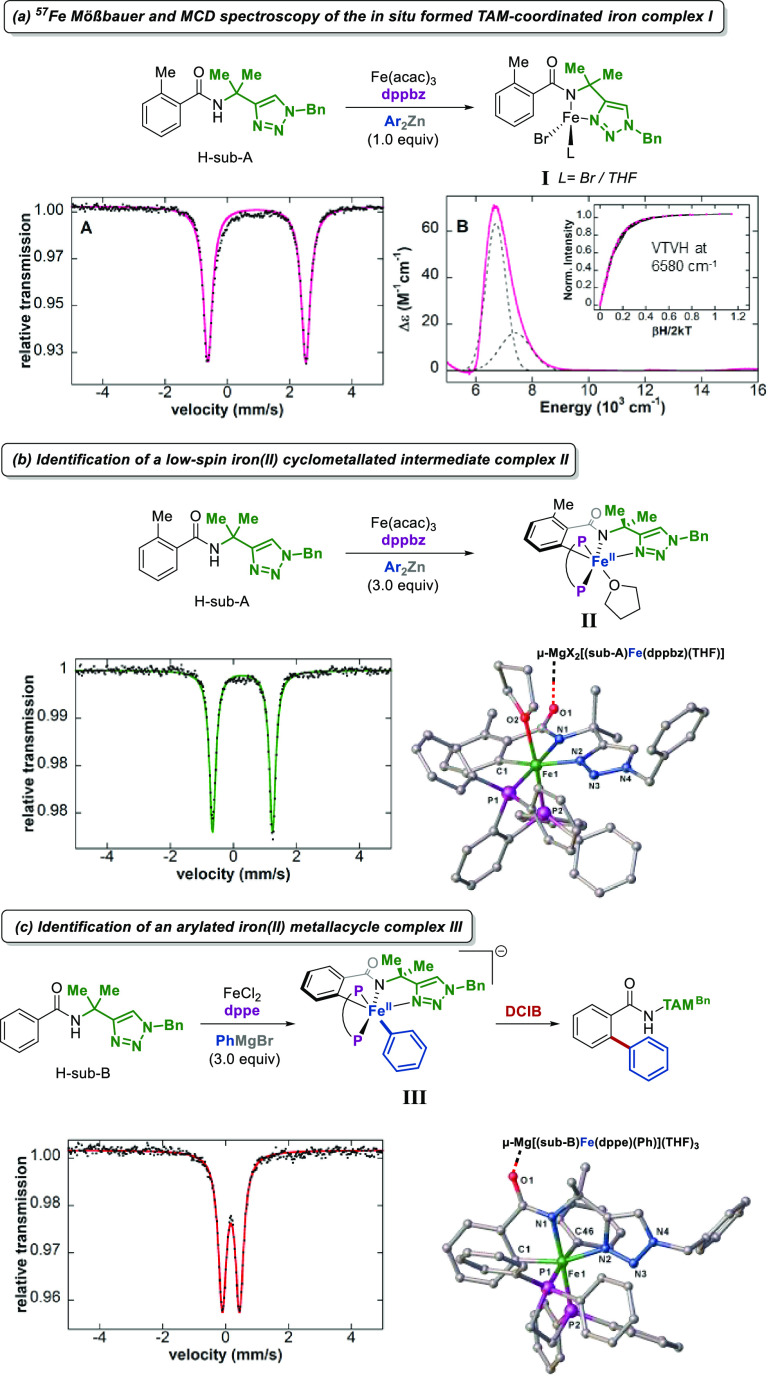
Synthesis and characterization of catalytically relevant cyclometalated iron species in TAM-directed iron-catalyzed C–H arylations. (a) ^57^Fe Mößbauer and MCD spectroscopy of the *in situ* formed iron species. (b) Synthesis of a catalytically active low-spin iron(II) intermediate. (c) Identification of an arylated iron(II) complex.

Subsequently, and in collaboration with Neidig,^34^ we embarked on a journey to understand the mode of operation of the TAM-assisted C–H activations aiming to rationalize the role of the reaction components (Grignard, oxidant, zinc additive) and understand the observed switch in reactivity from arylation^24^ to allylation/alkylation^30^ when allyl/alkyl halides are used as substrates in the absence of DCIB and zinc salts. Interestingly, analogous cyclometalated iron intermediates can form in both systems despite the use of different phosphines and arylating reagents according to mechanistic studies involving ^57^Fe Mößbauer spectroscopy, along with single-crystal X-ray crystallography and stoichiometric reactions (Figure 2).

**Scheme 3:**
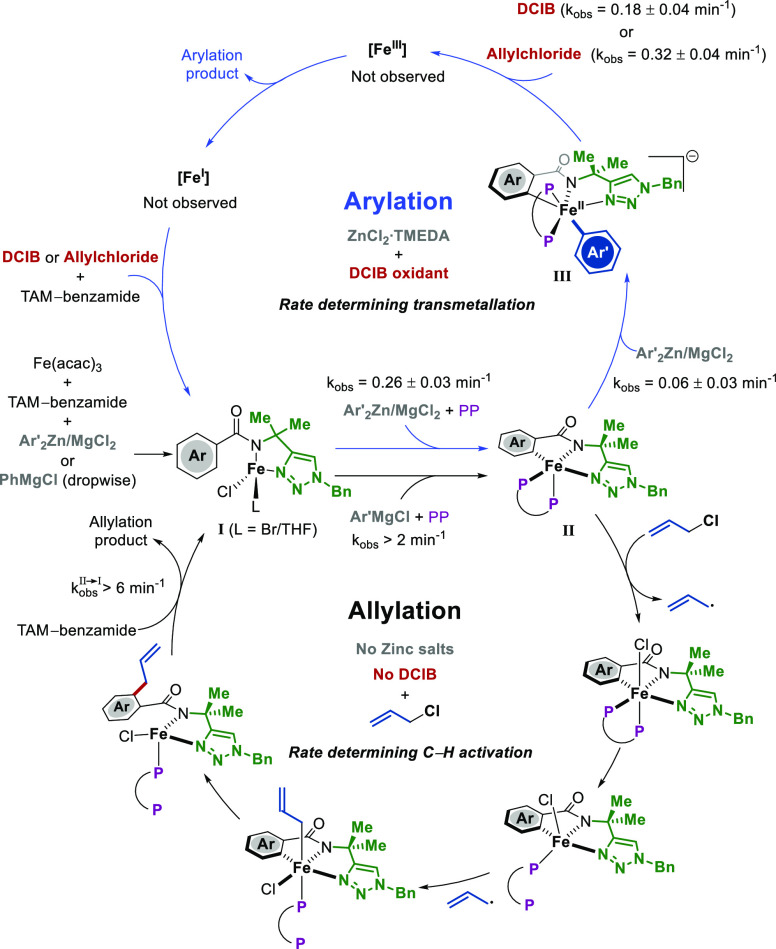
Catalytic Cycle for the TAM-Assisted Iron-Catalyzed C–H Arylation and Allylation

Therefore, simple TAM-benzamides were reacted with varying amounts of Grignard or aryl zinc reagents in the presence of iron salts and dppe or dppbz, leading to the isolation and complete characterization of iron complexes **I**–**III** as potential intermediates (Figure 2). Subsequently, their presence during catalysis was confirmed using ^57^Fe Mößbauer spectroscopy by analyzing freeze-trapped solution samples from catalytic reaction mixtures. The roles of **I**–**III** in catalysis were delineated by probing their reactivity in combination with kinetic studies. Notably, intermediate **II** represents the first structurally characterized, cyclometalated iron species shown to form under stoichiometric and catalytic conditions in directed iron-catalyzed C–H arylations and allylations with triazole assistance (Figure 2b). In addition, an arylated cyclometalated low-spin iron(II) analogue of complex **II**, intermediate **III**, was found to be responsible for the key C–C bond-forming step in arylations enabled after oxidation by DCIB at a rate consistent with catalysis (Figure 2c). Apart from the complexes **I**–**III**, low valent iron species were also detected, presumably resulting from over-reduction by the aryl zinc reagent. It was shown that these species could be reoxidized by DCIB and thus re-enter the catalytic cycle. Lastly, it was demonstrated that for arylations in the presence of zinc salts and DCIB, C–H activation is facile, with transmetalation being rate-determining and a Fe(II)/Fe(III)/Fe(I) catalytic cycle being operative in line with previous computational studies on the relevant quinoline system (Scheme 3).^35^

Upon replacing DCIB with allyl chloride, now acting as a substrate and oxidant, and removing the zinc salts, a complete shift in reactivity is observed from arylation to allylation. Detailed mechanistic studies by Neidig revealed that the two systems are interlinked by sharing intermediates **I** and **II** in their catalytic cycles (Scheme 3).^36^ Hence, in the absence of zinc, complex **II** can quickly react with allyl chloride to form allylated TAM-benzamides via an inner-sphere radical process involving a partial iron-bisphosphine dissociation, as inferred by DFT computational studies. This time, in contrast to the C–H arylation reaction,^34^ C–H activation is the rate-determining step due to the high reaction rate between complex **II** and allyl chloride, which favors allylation over arylation (note that excess Grignard reagent is still present during allylation). Interestingly, if zinc salts are added to the allylation system, a complete shift in reactivity back to arylation is observed even without DCIB since the allyl chloride can act as the oxidant. This role of zinc does not seem to be fully understood yet, possibly due to the complexity of the effect of zinc salts on iron catalysis.^37^

**Scheme 4:**
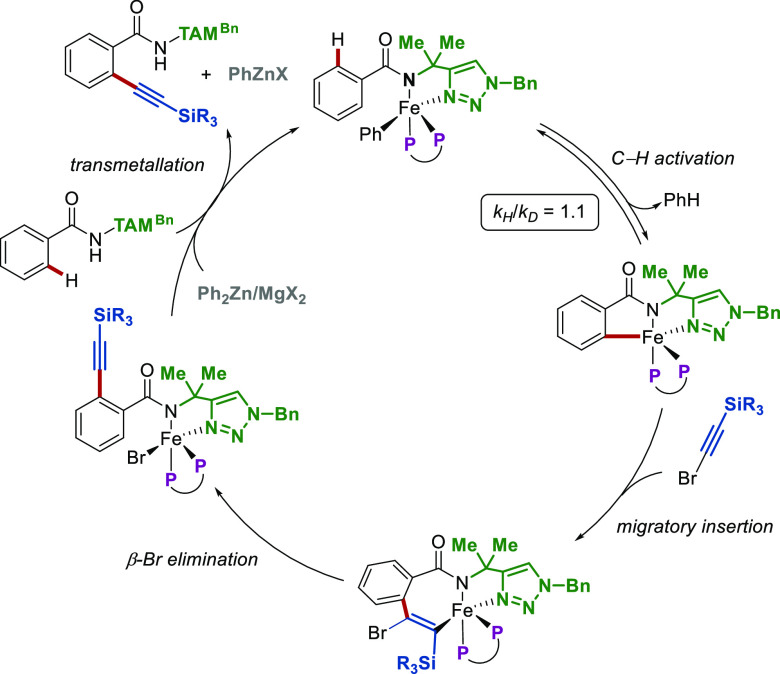
Proposed Mechanism for the Iron-Catalyzed C–H Alkynylation of TAM-Benzamides

Following arylations and alkylations, mechanisms have been proposed for the alkynylations^31^ and hydroarylations^32^ described in Scheme 2. Although the mechanistic studies on these systems were not as detailed, with only deuterium and competition experiments performed, reasonable catalytic cycles were suggested based on the arylation^34^ and allylation findings.^36^ Hence, according to intermolecular competition experiments and reactions with radical scavengers, a deprotonative σ-bond metathesis (DBM) elementary step was proposed for alkylations, excluding a single-electron-transfer (SET)-type mechanism. Thus, the catalytic cycle presented in Scheme 4 was proposed, commencing with the coordination of the substrate on the iron through TAM, followed by alkylation and reversible C–H activation. Finally, the resulting intermediate undergoes migratory insertion of the bromoalkyne to the C–Fe bond with subsequent β-Br elimination and transmetalation with the benzamide to give the desired product and regenerate the catalyst. A similar mechanistic scenario applies for hydroarylations,^32^ again involving a migratory insertion of the alkene to the C–Fe bond followed by transmetalation with zinc and product release after acidic workup. In both cases, C–H activation was not rate-limiting, according to the lack of any kinetic isotope effect observed.

### 3.3 Alkene and Alkane C–H Activations

**Scheme 5:**
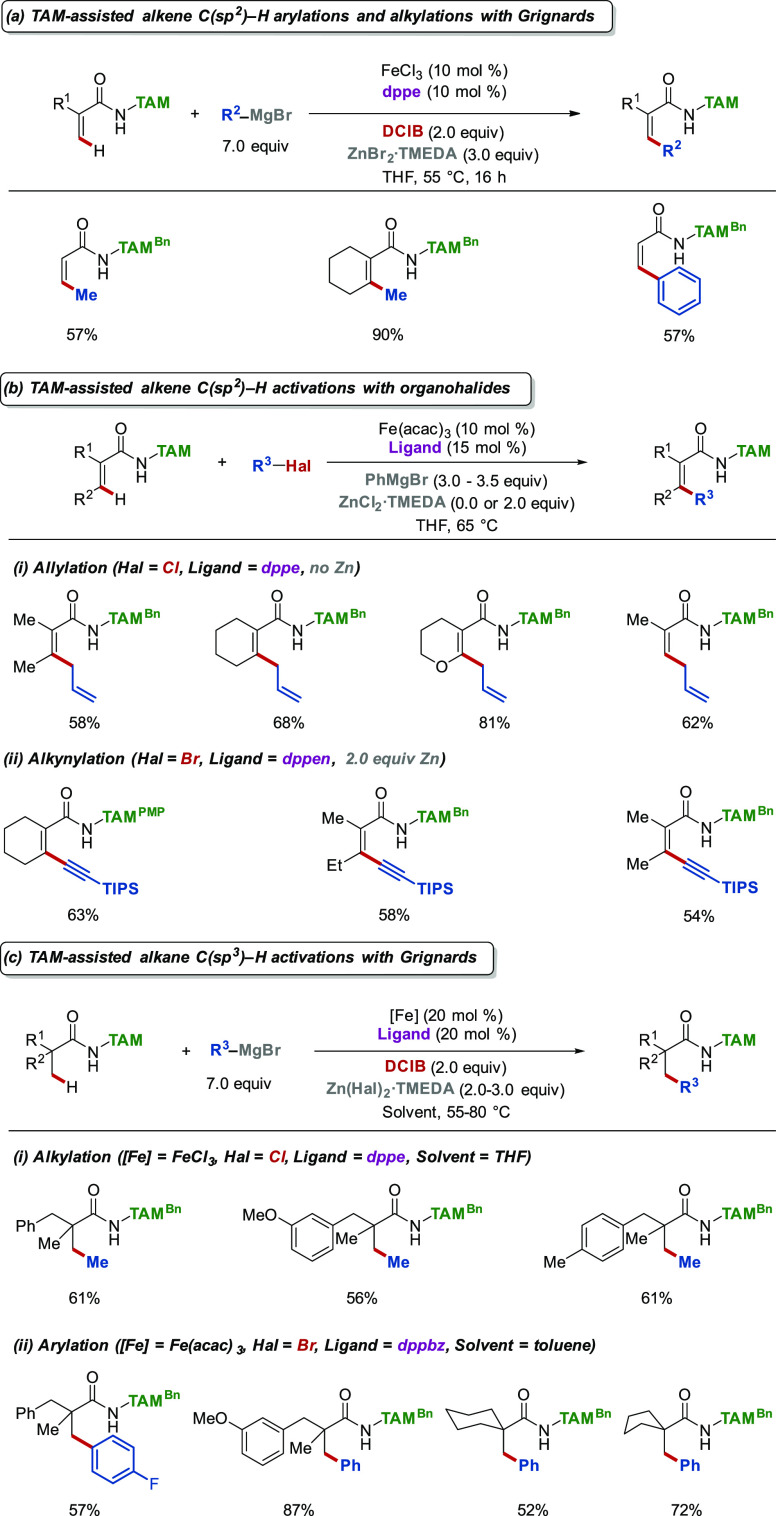
Summary of TAM-Directed Iron-Catalyzed Activations of Alkene and Alkane C–H Bonds

Olefins are versatile building blocks in organic synthesis and common structural motifs in molecules of interest. While the direct modification of readily available alkenes is in high demand, the selective olefinic C–H activation comes with the intrinsic challenge of controlling the site- and stereoselectivity.^7^ In this regard, C–H arylation and methylation under our developed TAM-assisted iron catalysis proved viable for alkenes with only the thermodynamically less stable *Z*-products formed (Scheme 5a).^24,25^ Similarly to alkylations and arylations, allylations^30^ and alkynylations^31^ were also possible with TAM-alkene substrates with high levels of stereoselectivity (Scheme 5b).

While several examples of iron-catalyzed C(sp^2^)–H bond activations have been presented so far, the activation of C(sp^3^)–H bonds continues to be challenging, with only limited strategies for direct C(sp^3^)–H methylations being reported.^19^ The difficulty in achieving such transformations can be attributed to the low polarity of the C(sp^3^)–H bonds in combination with the low Fe–C(sp^3^) bond strength. In addition, the absence of the arene prevents any potential initial π-coordination. Nevertheless, C(sp^3^)–H activations are essential, since the homologation of carbon chains provides a valuable tool for studying structure–activity relationships for drug development. Therefore, we harnessed triazoles for unprecedented iron-catalyzed C(sp^3^)–H arylations^24^ and methylations^25^ (Scheme 5c).

### 3.4 Annulation Reactions

Isoquinolones represent a privileged structural motif in various biologically active molecules of medicinal interest. Their synthesis can be accessed directly via the transition-metal catalyzed C–H annulation of benzamides with substrates containing multiple C–C bonds.^7,17^ Despite indisputable advances in directed C–H annulation toward isoquinolone construction, a benign iron-catalyzed strategy facilitated by peptide isosteres, such as triazoles, has proven elusive. Here, the diversified synthesis of isoquinolones was achieved through oxidative alkyne annulations,^38^ redox-neutral alkyne^39^ and allene annulations,^40^ and bicyclopropylidene (BCP) annulations.^1^

**Scheme 6:**
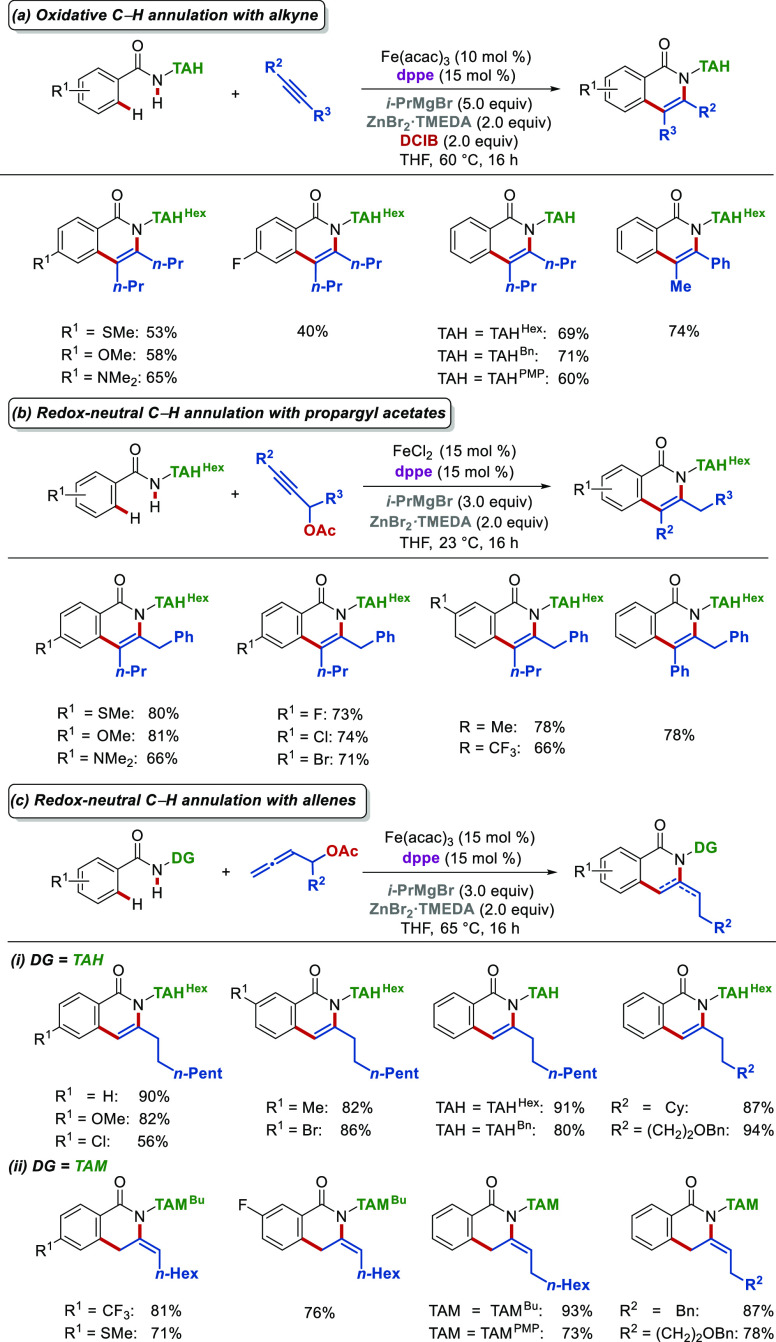
Triazole-Directed Iron-Catalyzed C–H Annulations with Alkynes and Allenes

To access highly decorated isoquinolones, an iron-catalyzed oxidative C–H/N–H functionalization strategy was developed (Scheme 6a).^38^ In analogy to TAM-assisted C–H arylations (Scheme 2), DCIB was required, while complete regioselectivity was observed with methyl-aryl alkyne substrates. The observed regioselectivity can be attributed to repulsive steric interactions arising from the compact nature of the iron intermediates that force the alkyne to coordinate stereospecifically during the key migratory insertion step. When TAH was replaced by AQ, a 3-fold decrease in yield was observed due to the lack of structural flexibility in the latter.^41^ Nevertheless, a fundamental limitation of this iron(II)-catalyzed C–H annulation strategy is using toxic and costly DCIB oxidant (*vide supra*).^38,41^ To circumvent this problem, we developed a redox-neutral annulation strategy where DCIB is replaced by C–O bonds acting as an internal oxidant in easily accessible propargyl^39^ and allenyl^40^ acetates (Scheme 6b). Importantly, higher yields were achieved with redox-neutral annulations^39,40^ compared to oxidative annulations^38^ (Scheme 6, part a vs part b) with the challenging *meta*-substituted benzamides, which were inactive under oxidative conditions,^38^ being converted in a highly regioselective fashion. In addition, our redox-neutral methodology enabled the conversion of Cl- and Br-substituted benzamides, which was impossible under oxidative annulation conditions.^38^ This increased reactivity with propargyl/allenyl acetates can be attributed to the higher efficiency of intramolecular electron transfer over that of the intermolecular one. Lastly, distinct chemoselectivities were achieved by the judicious choice of the directing triazole. Hence, a more flexible TAH directing group led to isoquinolones, while the TAM group favored the formation of non-aromatic *exo*-methylene dihydroisoquinolones (Scheme 6c).

**Scheme 7:**
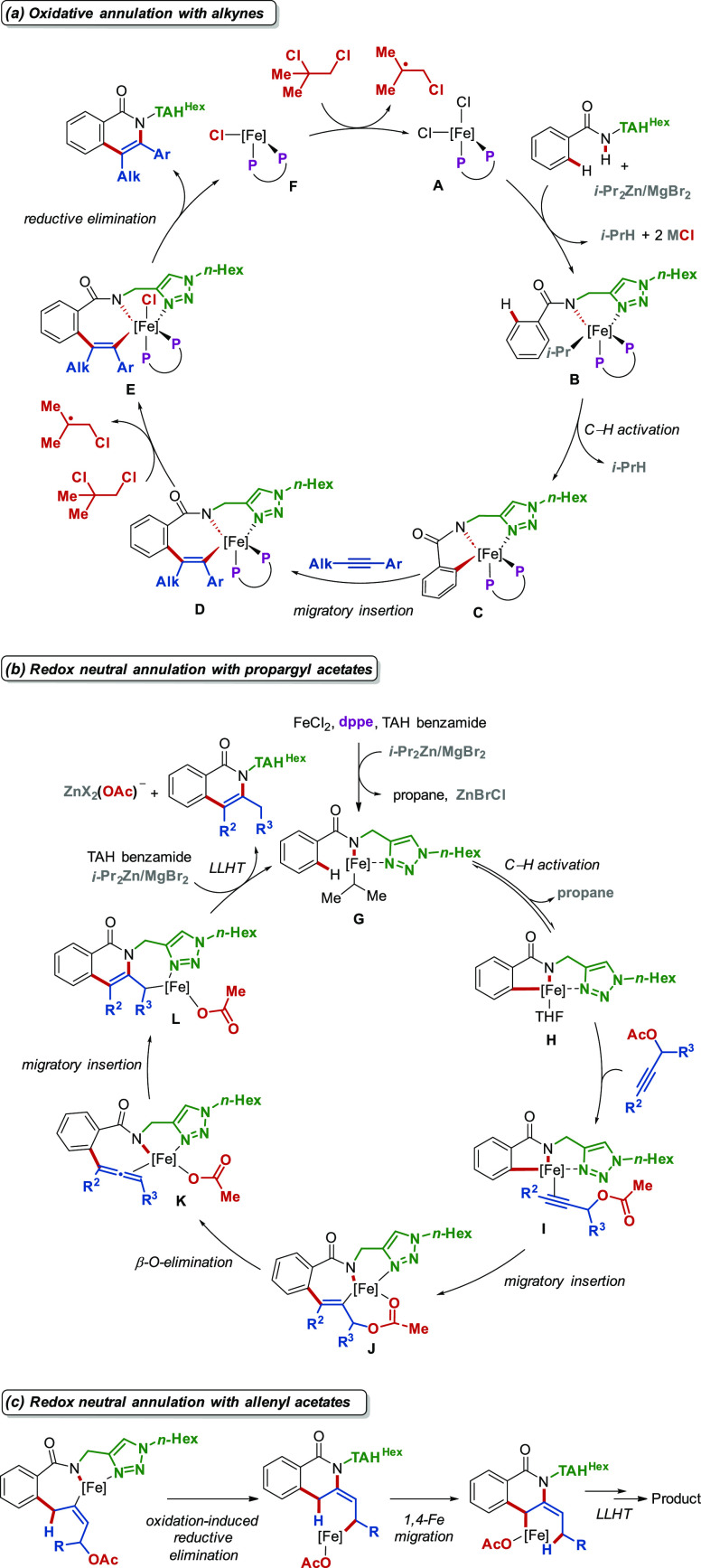
Proposed Mechanisms for Oxidative and Redox-Neutral Annulations

Regarding the mechanism, in both oxidative and redox-neutral annulations, C–H activation on an iron(II) center occurs as the first step, followed by the migratory insertion of the second substrate to form a key 7-membered metallacycle intermediate. From this point on, the fate of this metallacyclic species differentiates the two processes. In oxidative annulations (Scheme 7a), intermediate **D** gets oxidized by DCIB to iron(III) complex **E**, which can then reductively eliminate to release the product. In contrast, the redox-neutral annulation (Scheme 7b) proceeds through β-O-elimination followed by a migratory insertion of iron-coordinated allene intermediate **K** to give intermediate **L**. Subsequent LLHT completes the catalytic cycle and delivers the product. The two mechanisms were delineated via experimental techniques, such as reactions with radical scavengers, Mößbauer spectroscopy in collaboration with the Meyer group, Hammett-plot analysis, deuterium labeling experiments, and DFT calculations. Lastly, when allenyl acetates are employed, the proposed mechanism is similar to the annulation with propargyl acetate but also includes a rare 1,4-Fe migration regime (Scheme 7c), which was also observed later in an iron-catalyzed C–H alkylation of aromatic ketones.^42^

**Scheme 8:**
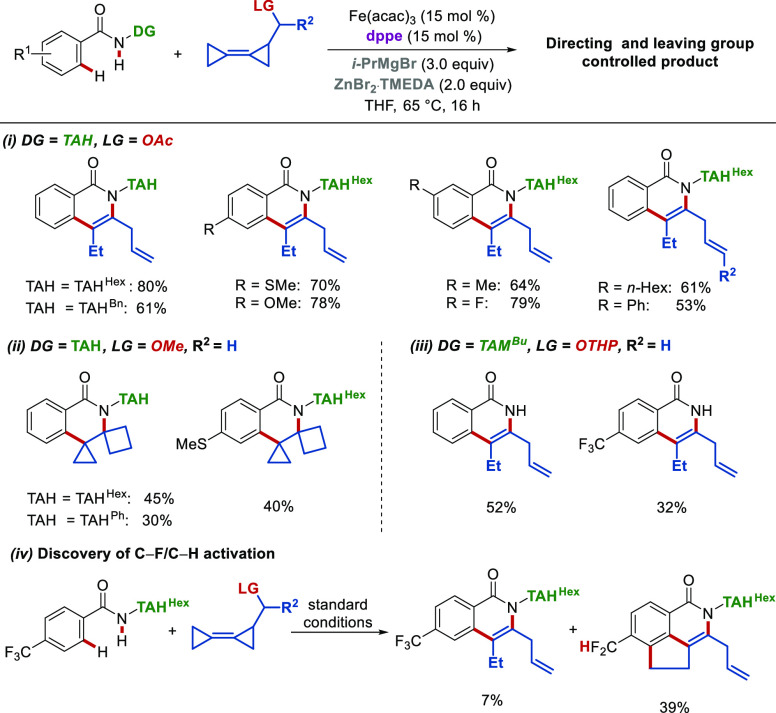
Iron-Catalyzed C–H Annulations with BCPs

Following simple alkynes and allenes, more structurally complex substrates, such as bicyclopropylidenes (BCPs), were explored.^1^ BCPs are unique since they exhibit reactivity beyond typical C–C double bonds due to their ring strain. Therefore, we reported the first iron-catalyzed C–H/C–C activation with BCPs by combining sustainable C–H/C–C activation with BCP chemistry. Three distinct products could be chemoselectively obtained through the judicious choice of the directing and leaving groups (Scheme 8). Combining TAH as the directing group and acetate as the leaving group yielded isoquinolones in high yields and regioselectivity. In contrast, spiro-fused isoquinolones were formed in TAH-assisted iron-catalyzed C–H annulations, exploiting methoxy as a leaving group, albeit in moderate yields. With TAM as an auxiliary, cleavage of the directing group occurred under the reaction conditions through β-C elimination-mediated C–N cleavage between the TAM and the benzamide, affording free isoquinolones. Notably, a rare C–F/C–H activation was observed for the first time in iron catalysis when the CF_3_- *para*-substituted TAH-benzamide was employed, providing the C–H/C–C/C–F/C–H functionalized product (Scheme 8iv).

**Scheme 9:**
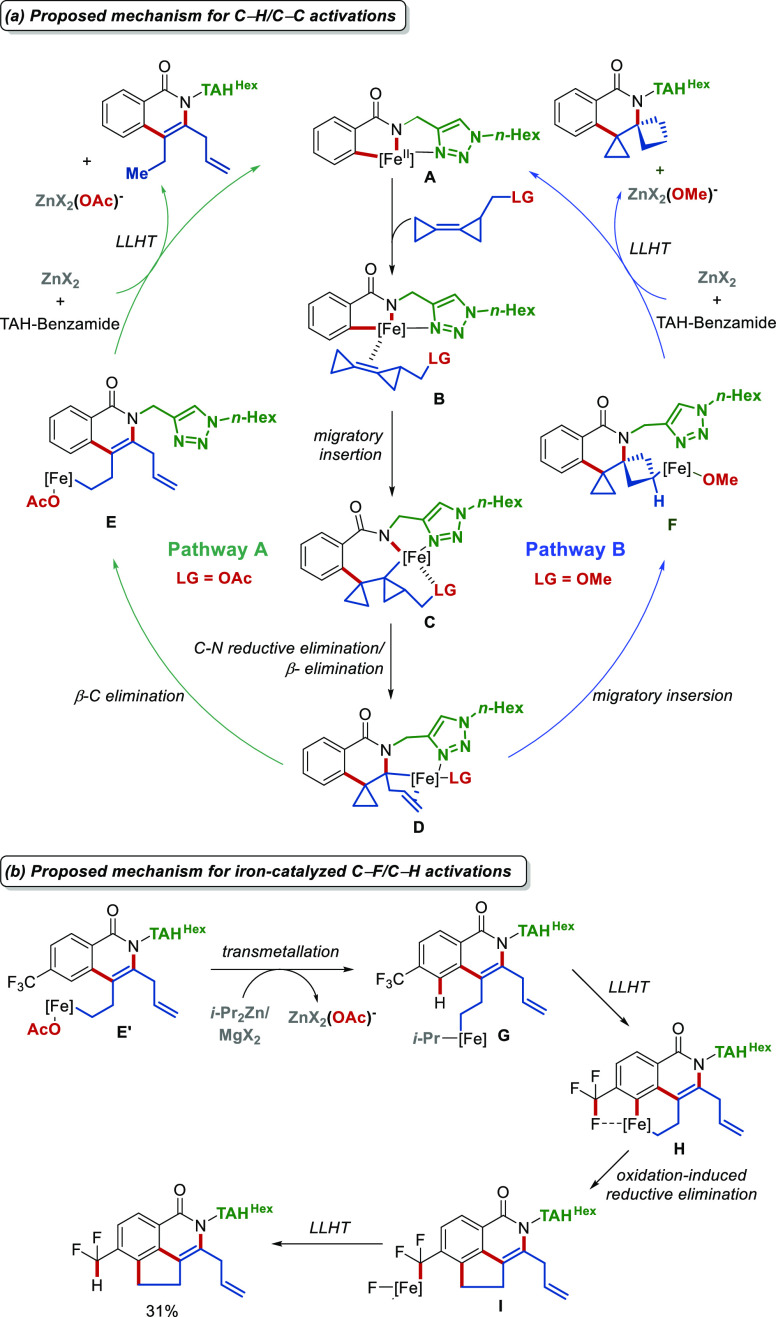
Proposed Catalytic Cycle for Iron-Catalyzed C–H Annulations with BCPs

The mechanism for these annulations with BCPs is bifurcated, with the bifurcation point being intermediate **D**, which is formed by C–N reductive elimination and β-C elimination of the key seven-membered intermediate **C** (Scheme 9a). Hence, the cyclopropyl group of intermediate **D^OAc^** (LG = acetoxy) favors a β-C elimination toward intermediate **E** (Pathway A, Scheme 9a). In contrast, alkene migratory insertion occurs in **D^OMe^** toward intermediate **F** with methoxy as the leaving group (Pathway B, Scheme 9a). In addition, we proposed the novel C–F/C–H activation sequence to proceed through the oxidation-induced reductive elimination of intermediate **H** (Scheme 9b). Deuterium labeling experiments and Mößbauer spectroscopic studies of catalytic reaction mixtures experimentally supported our proposed mechanisms.

## 4 Ferraelectro-Catalyzed C–H Activation

**Scheme 10:**
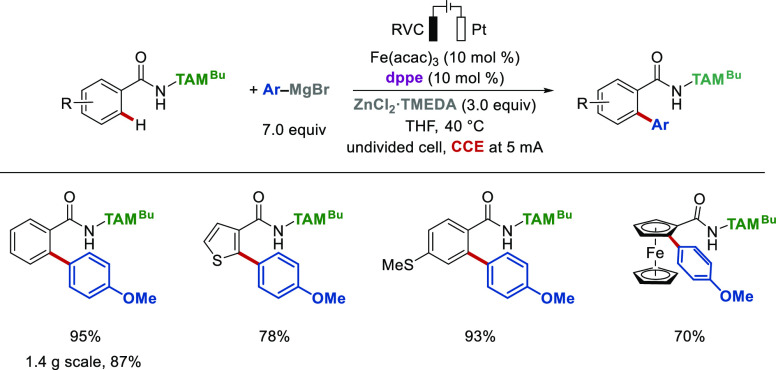
Iron-Catalyzed Electro-Oxidative C–H Arylations

A fundamental limitation of the thus far available iron-catalyzed C–H activations is the necessity for oxidants such as overstoichiometric DCIB or prefunctionalization with acetate leaving groups in the substrates acting as an internal oxidant. The high cost of DCIB of >7500 €/mol and its highly flammable and corrosive nature impedes its use on a larger scale.^2^ This limitation was circumvented by exploiting electricity as an environmentally benign and cost-effective oxidant, enabling sustainable ferraelectro-catalyzed arylations (Scheme 10).^2^ The iron electrocatalysis tolerated oxidant-sensitive sulfides and was performed at a gram scale, outperforming the DCIB-mediated reaction, featuring a user-friendly setup. Notably, C–H arylation proceeded efficiently without supporting electrolyte, largely due to the presence of magnesium and zinc salts.

**Figure 3:**
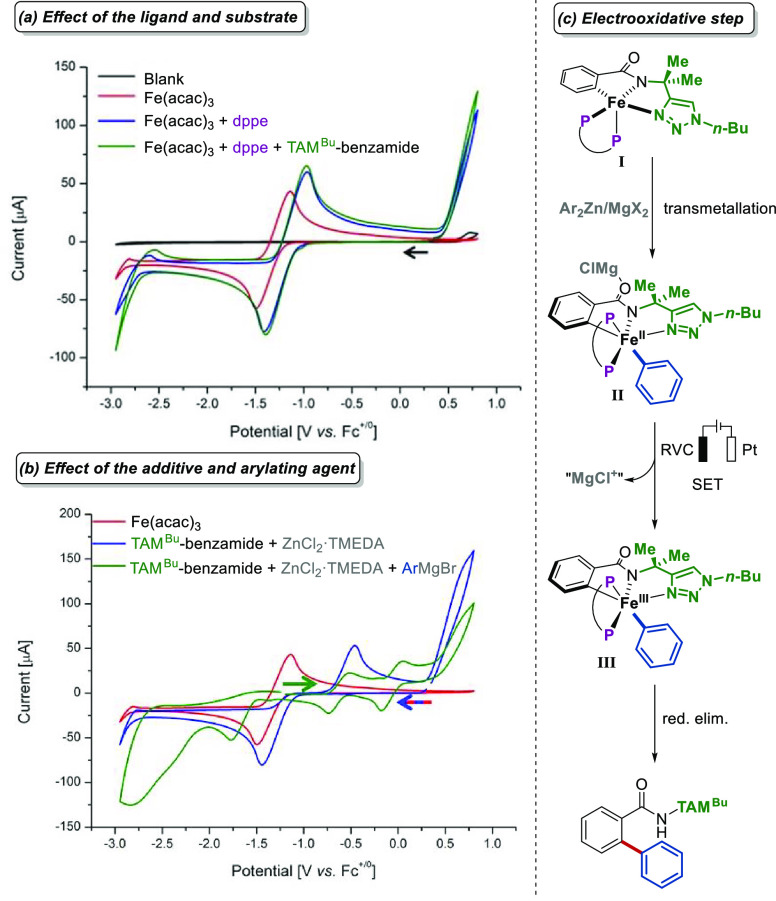
Cyclic voltammetric studies of the iron-catalyzed electrooxidative C–H arylation reaction and its proposed electrooxidative step.

From a mechanistic perspective, cyclic voltammetric studies suggest that coordination of dppe to Fe(acac)_3_ occurs (Figure 3a). In addition, two new reversible redox events were observed upon reaction with ArMgBr (iron(I)/iron(II) and iron(II)/iron(III), Figure 3b) which align well with our computational findings on iron(II/III/I) catalysis and the cyclic voltammetric studies reported by Jutand^43^ on iron-catalyzed Kumada–Corriu type cross-couplings, as well as previous Mößbauer spectroscopic studies on triazole-assisted iron-catalyzed C–H arylation (Scheme 3).^34^ DFT calculations shed light on the nature of the electrooxidative step (Figure 3c) with the most favored pathway involving transmetalation facilitated by coordination with MgCl^+^ to form a bimetallic iron intermediate **II**, which leads via anodic oxidation to the aryl-iron(III) complex **III**. The calculated half-wave oxidation potential associated with this process is 0.01 V vs. ferrocene, which aligns well with experimental values (*vide supra*). This bimetallic intermediate is also in agreement with the cyclometalated iron complexes presented in Figure 2.

## 5 Low-Valent Iron-Catalyzed C–H Activations by Weak *O*-Coordination

The transformations described herein, albeit iron-catalyzed, require large amounts of the correct combination of external oxidants as well as magnesium- and zinc-based organometallic reagents. These characteristics lower the sustainable nature of such methodologies by generating large amounts of byproducts and reducing the atom economy while increasing the complexity of the reaction setup.

**Scheme 11:**
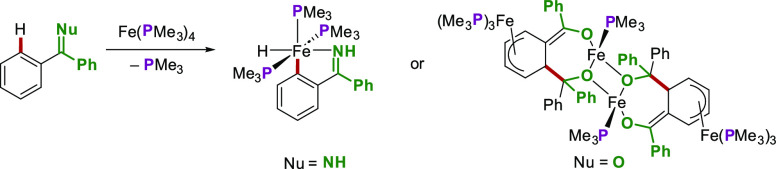
Examples of Stoichiometric C–H Activations of Imines and Ketones with Low-Valent Iron Complexes

To circumvent these problems and develop sustainable C–H activations, low-valent iron complexes were employed as single precatalysts without additives. The resulting C–H activation also has the added advantage of not requiring designed directing groups with the weakly coordinating oxygen carbonyl atom being sufficient. The use of iron(0) complexes in stoichiometric C–H activations is well documented: in early reports, low-valent iron species were demonstrated to oxidatively add to the C–H bonds of benzophenones^44^ and ketimines,^45^ revealing the potential for development of innovative catalytic C–H activation processes (Scheme 11).

**Scheme 12:**
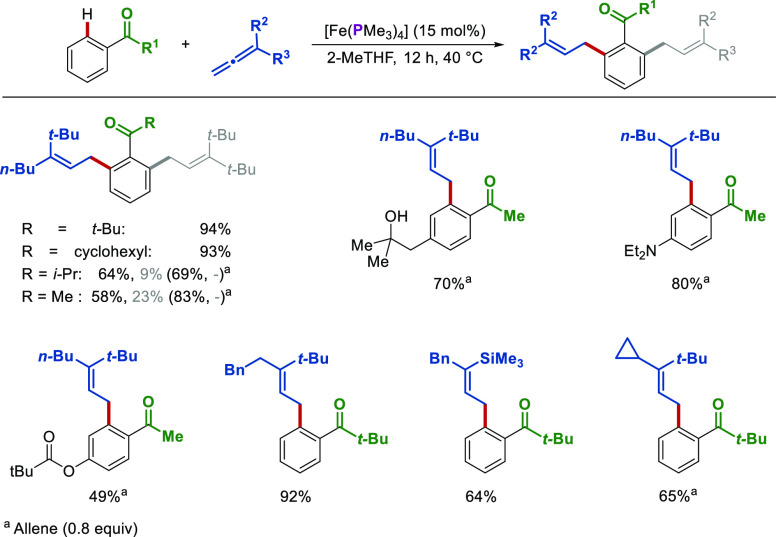
Iron-Catalyzed C–H Activation by Weak *O*-Coordination

Hence, the Fe(PMe_3_)_4_-catalyzed hydroarylation of alkenes was described by Kakiuchi,^42,46^ while we reported the first low-valent iron-catalyzed C–H allylation^3^ accompanied by detailed mechanistic studies.^47^ The catalysis proceeded in biomass-derived 2-MeTHF with [Fe(PMe_3_)_4_] as a single component precatalyst with chemoselectivity being driven by steric effects of the directing group (Scheme 12).^3^ Notably, a broad substrate scope and good functional group tolerance were observed, with sensitive groups such as hydroxyl, amino, and alkoxycarbonyl being well tolerated (Scheme 12).

**Scheme 13:**
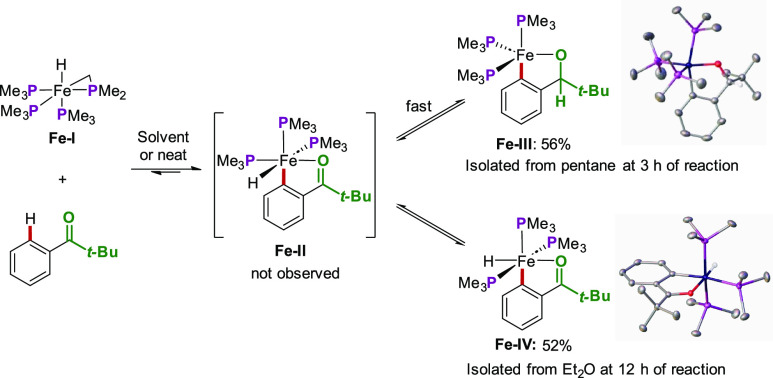
Isolation of Cyclometalated Iron Complexes in Allene Hydroarylations

The mechanism of this allene hydroarylation was initially probed by performing stoichiometric reactions between [Fe(PMe_3_)_4_] and the pivalophenone substrate (Scheme 13). This led to the isolation of two key iron cyclometalated intermediates: an iron alkoxide complex (**Fe-III**) and a *mer* iron hydride complex (**Fe-IV**). The two complexes were in equilibrium, interconverting via a *fac*-iron hydride species (**Fe-II**). The latter could not be observed or isolated and was therefore confirmed via DFT calculations.^47^

**Figure 4:**
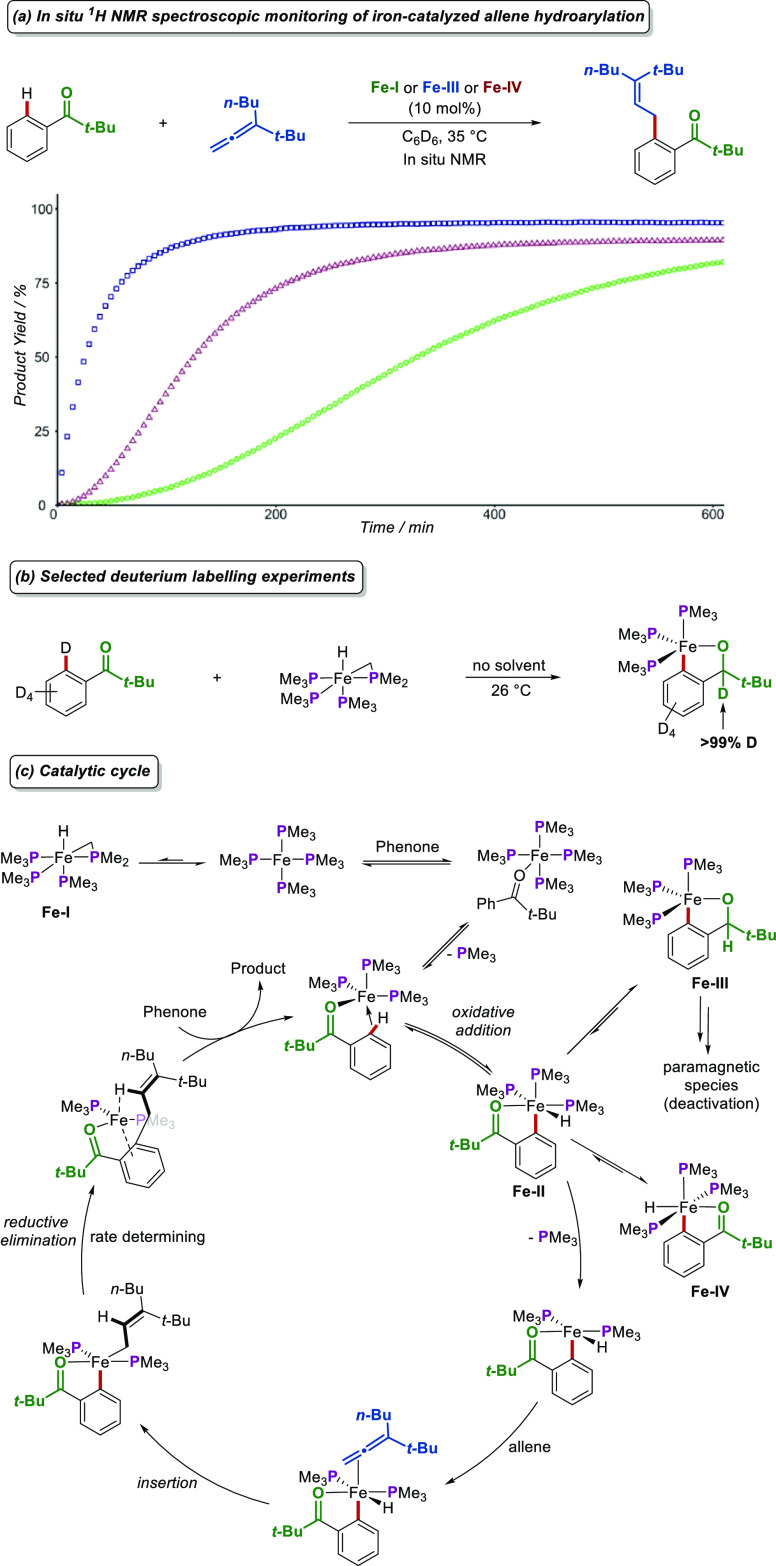
Key mechanistic experiments on iron-catalyzed allene hydroarylations and the proposed catalytic cycle.

Subsequently, temporal plots of the reaction’s progress were obtained using complexes [Fe(PMe_3_)_4_] (**Fe-I**), **Fe-III**, and **Fe-IV** as precatalysts (Figure 4a).^47^ An induction period was observed with complexes **Fe-I** and **Fe-IV**, excluding them from being on the cycle species. In contrast, the alkoxide complex **Fe-III** catalyzed the reaction with the highest rate and without an induction period. Nevertheless, alkoxide **Fe-III** cannot yet be considered on-cycle, since it could be in a fast equilibrium with the true on-cycle species, such as **Fe-II**, which could not be isolated. Hence, DFT calculations provided support for the most energetically favored mechanistic pathway involving the *fac* iron hydride complex **Fe-II**. Based on further EPR and *in situ* NMR spectroscopy, stoichiometric experiments, kinetic studies, and deuterium labeling experiments (Figure 4b), the mechanism described in Figure 4c was obtained.

The proposed mechanism aligns with all experimental and computational data, with C–H activation occurring via a barrierless, reversible oxidative addition of the phenone to an Fe(0) intermediate toward the *fac*-iron hydride complex **Fe-II** which can isomerize to **Fe-IV**, β-hydride eliminate toward alkoxide **Fe-III**, or coordinate with the allene after phosphine decoordination. Subsequent migratory insertion, followed by rate-limiting reductive elimination and ligand exchange, regenerates the active catalyst and yields the desired product. Catalyst deactivation pathways toward paramagnetic complexes were also considered according to EPR spectroscopic investigations. The obtained mechanistic understanding will lead to the development of new sustainable additive-free low-valent iron C–H activations, advancing this currently emerging and exciting field.

## 6 Iron-Catalyzed Enantioselective C–H Activation

The importance of developing enantioselective catalytic transformations is well established.^8^ Chiral compounds are omnipresent in nature, and predominantly, only one enantiomer exhibits the desired properties. Therefore, considering the sustainability advantages of iron-catalyzed C–H activations, the scarcity of enantioselective versions of these reactions is surprising. However, this reflects the challenging nature of this field and the need to intensify our efforts for its development.

**Scheme 14:**
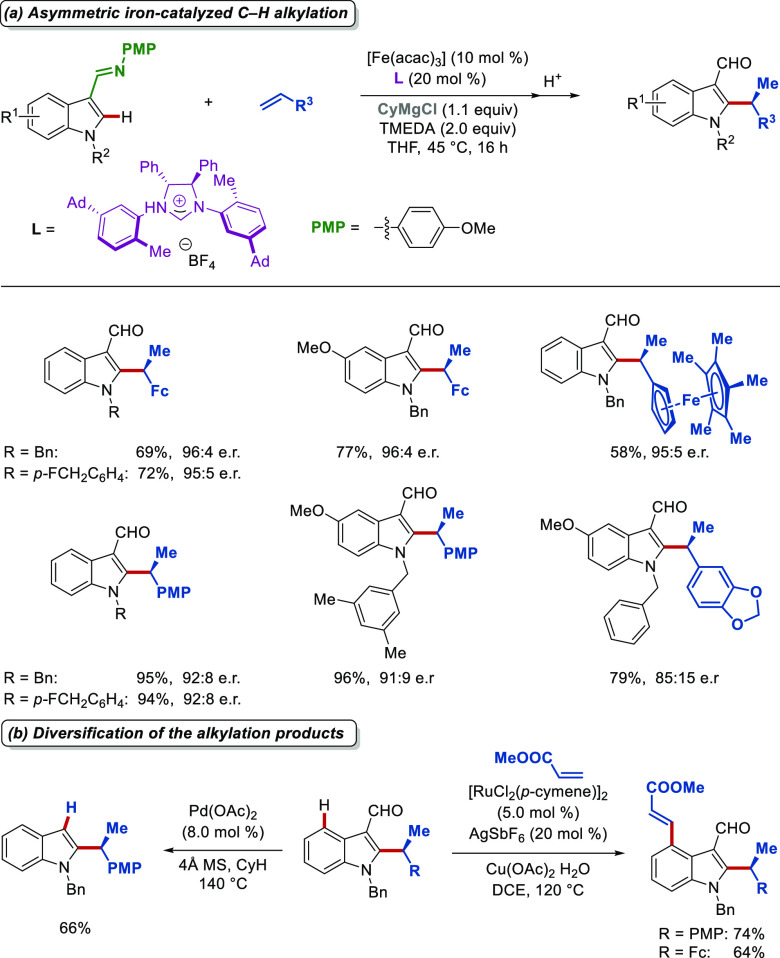
Enantioselective Iron-Catalyzed C–H Activation

To this end, we reported the first highly efficient enantioselective iron-catalyzed C–H activation using a specially designed *N*-heterocyclic carbene ligand for the C2-alkylation of (aza)indoles (Scheme 14a).^4^ The imine motif could be easily removed with acidic workup, and the resulting chiral aldehydes were further diversified in an expedient manner (Scheme 14b).

**Figure 5:**
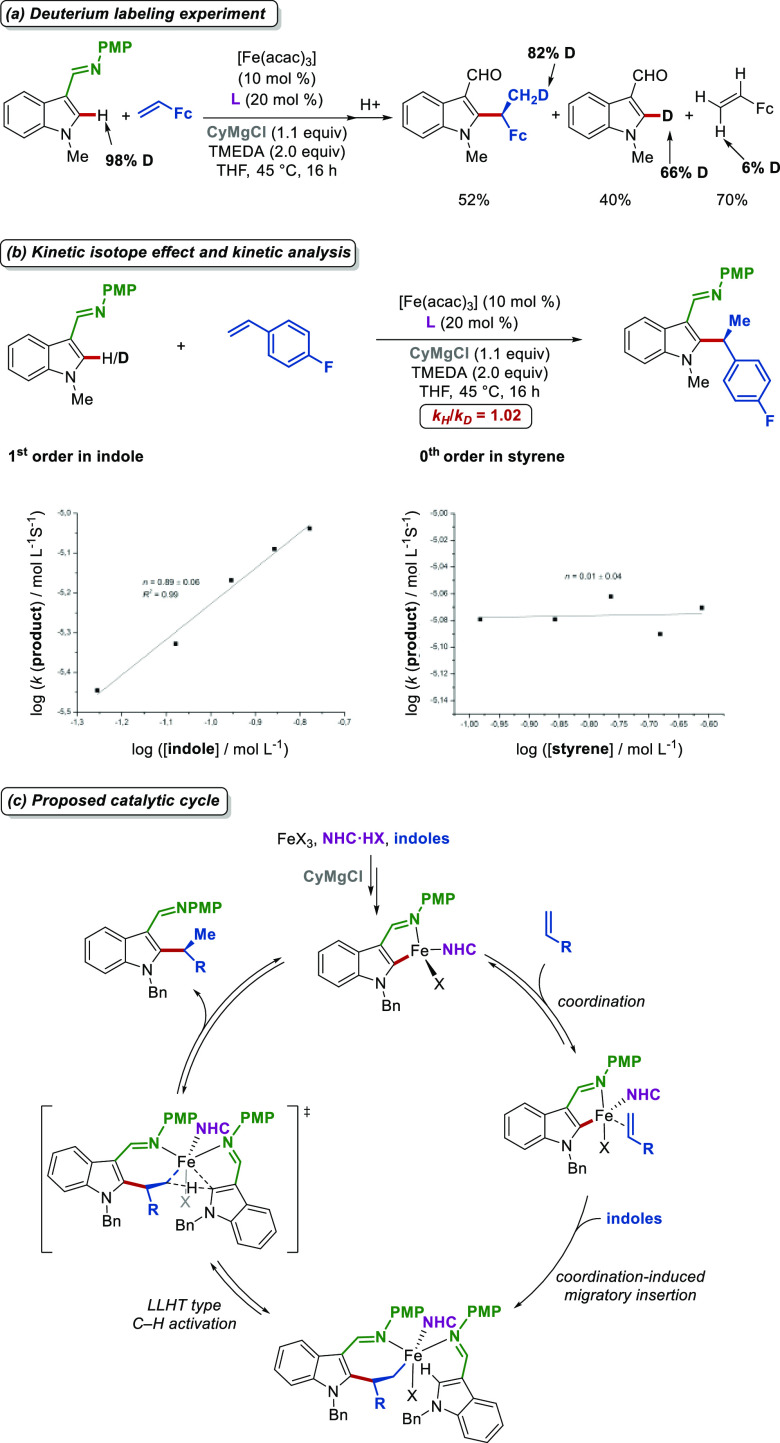
Key mechanistic experiments and proposed mechanism in enantioselective iron-catalyzed C–H activation.

From a mechanism viewpoint, C–H alkylations with isotopically labeled substrates support an inner-sphere C–H activation mechanism (Figure 5a). In addition, a LLHT pathway was proposed for the C–H cleavage step according to deuterium labeling experiments and kinetic studies (Figure 5b). Mößbauer spectroscopic and electrospray-ionization mass spectrometric studies were conducted to gain further mechanistic insights.^48^ Hence, an iron(II)-NHC species was proposed as being catalytically active. According to these mechanistic findings, the catalytic cycle presented in Figure 5c was proposed for this iron-catalyzed asymmetric C–H activation.

## 7 Conclusion

The current geo-economical situation calls for the design of sustainable catalysis manifolds, translating into a strong need for inexpensive and nontoxic 3d transition metals to replace precious and toxic iridium, palladium, ruthenium, and rhodium catalysts. Iron stands on top in terms of sustainability due to its low cost, nonexistent toxicity, and high natural abundance. Therefore, recent years have witnessed significant progress in iron-catalyzed C–H activations. As part of this continuing effort, peptide isosteric triazoles emerged as site-selective guaranteeing motifs for iron-catalyzed C–H transformations. The modular nature of these triazoles is relevant to medicinal chemistry and proved to be instrumental for oxidative C–H transformations proceeding through C–H, C–H/C–C, or C–H/Het–H bond cleavages. Elegant experimental and computational mechanistic studies were the key to success, providing essential insights into the valence and spin state of catalytically active iron complex intermediates and highlighting the crucial role of the phosphine ligands in controlling SET with organoiron species. Given the sustainable nature of both C–H activation and iron catalysis, further advances are expected in this rapidly evolving arena, including *inter alia* additive-free iron-catalyzed C–H activations, the use of weakly coordinating directing groups, enantioselective iron-catalyzed C–H activations for multiple stereogenic centers, and ferraelectro-catalysis, which likely will be based on detailed mechanistic understanding.
